# Fluorescent Property of Chitosan Oligomer and Its Application as a Metal Ion Sensor

**DOI:** 10.3390/md15040105

**Published:** 2017-04-04

**Authors:** Hun Min Lee, Min Hee Kim, Young Il Yoon, Won Ho Park

**Affiliations:** 1 Department of Advanced Organic Materials and Textile System Engineering, Chungnam National University, Daejeon 34134, Korea; hun1062@naver.com (H.M.L.); vvvkmhvvv@nate.com (M.H.K.); 2 Laboratory of Molecular Imaging and Nanomedicine (LOMIN), National Institute of Biomedical Imaging and Bioengineering (NIBIB), National Institutes of Health (NIH), Bethesda, MD 20892, USA

**Keywords:** chitosan oligomer, fluorescent property, metal ion sensor

## Abstract

An aqueous solution was successfully prepared using a low-molecular-weight chitosan oligomer and FITC, and its structural and fluorescent properties were observed by using ^1^H NMR, ^13^C NMR, FT-IR, XRD, UV-Vis, and PL spectrometry. Its application as a metal ion sensor was also evaluated. The fluorescence in the water-soluble chitosan oligomer was a result of the carbamato anion (NHCOO-), and a synthesized FITC-labeled chitosan oligomer exhibited an effective detection effect for copper ion as well as energy transfer by the ion near FITC that caused a fluorescence decrease (quenching). The chitosan oligomer was confirmed to be applicable as a selective and sensitive colorimetric sensor to detect Cu^2+^.

## 1. Introduction

Among all natural polymers, chitosan is a promising biopolymer that is commercially available. It is a well-known polysaccharide that is mainly produced from chitin [1], and in the past few decades, naturally occurring chitosan has attracted a significant amount of interest due to its large quantities in nature, biodegradability, and extensive applicability [2]. A water-soluble chitosan oligomer is composed of β-(1,4)-2-amido-2-deoxy-d-glucan and β-(1,4)-2-acetoamido-2-deoxy-d-glucan (acetylglucosamine), and a substance with a low molecular weight can be obtained through acidic or enzymatic hydrolysis of chitosan. To date, many researchers have examined chitosan oligomers as promising materials for biomedical applications due to their good biocompatibility, biodegradability, antimicrobial activity, and wound healing effects [3,4,5].

Dye-labeled chitosan can be also employed in bio-imaging systems because it has little toxicity. Some dyes, such as Alexa Fluor, Cibacron Blue, and fluorescein isothiocyanate (FITC), have been used to create a dye-labeled chitosan particulate system [6]. However, a significant part of such approaches uses chemical reagents in the synthesis of dye-labeled chitosan due to its low solubility in water [7]. Numerous studies have addressed the utilization of chitosan as a fluorescence probe [8,9]. This approach has limited applicability in medical and pharmaceutical applications because most of these may be environmentally toxic or biologically hazardous [10].

Copper ions (Cu^2+^) have been classified as a potentially carcinogenic substance because they induce DNA damages [11]. Malondialdehyde and 4-hydroxynonenal are produced through a reaction of the ions with lipid hydroxyperoxide, and this can result in impairment to tissues [12]. Therefore, effective detection of these ions is required for various fields [13].

This study focused on the fluorescent property of a low-molecular-weight chitosan oligomer and an environmentally friendly approach using a water-soluble chitosan derivative to sense the metal ion (Cu^2+^). The simple synthesis and fluorescent properties of the FITC-labeled chitosan oligomer were carried out in distilled water.

## 2. Materials and Methods

### 2.1. Materials

A chitosan (CHI) oligomer was provided by Kittolife Co., Pyeongtaek, Korea. Its degree of deacetylation (DD), molecular weight, and Cl ion content were 97%, ~1000 Da, and 3.2% respectively. Fluorescein isothiocyanate (FITC) was purchased from Sigma-Aldrich Co., Saint Louis, MO, USA. Ethanol (EtOH) was obtained from Samchun Chemical Co., Yeosu, Korea. Metal cations such as Na^+^, Cr^+^, Ni^+^, Sn^+^, Li^2+^, Mg^2+^, Al^2+^, Co^+^, Ni^2+^, Cu^2+^, Zn^2+^, Cd^2+^, Hg^2+^, Pb^2+^, and Fe^3+^ were supplied from Alfa Aesar Co., Haverhill, MA, USA. The chemicals were used without further purification or additional processes.

### 2.2. Preparation of CHI Oligomer-FITC Complexes

To conduct the one-step synthesis of CHI oligomer-FITC complexes, 100 mL solutions of 0.01%–0.05% (*w*/*v*) FITC in EtOH were added to a 10 mL solution of 2% (*w*/*v*) CHI oligomer in distilled water (DW). To obtain the desired products, the solutions were stirred at room temperature for 24 h in a darkroom. After the reaction, the unreacted FITC was removed through two centrifugal separations at 4000 rpm for 10 min using EtOH. The end products were obtained as a powder by using a vacuum dryer (VO-10x, Jeio Tech Co., Seoul, Korea) at room temperature for 24 h.

### 2.3. Structural Analyses of the CHI Oligomer and CHI Oligomer-FITC Complexes

To verify the presence of carbamato anion (NHCOO-) in the CHI oligomer, ^13^C nuclear magnetic resonance (^13^C NMR) spectra were recorded on a NMR spectrometer (300 MHz, FT-NMR, Bruker, Billerica, MA, USA). The Fourier transform vacuum infrared (FT-IR) spectra of the CHI oligomer-FITC complexes were collected using a FT-IR spectrometer (VERTEX 80v, Bruker) with a frequency range of 675–4000 cm^−1^, and X-ray diffraction (XRD) patterns were obtained at room temperature with 2θ = 5°–80° using an X-ray diffractometer (D8 DISCOVER, Bruker AXS, Billerica, MA, USA) to confirm structural changes in the complexes. Also, the degree of substitution (DS) of the complexes is ascertained by recording ^1^H nuclear magnetic resonance (1H NMR) spectra on a NMR spectrometer (300 MHz, FT-NMR, Bruker). The DS (%) values were expressed as (I_AR_/9)/(I_H2-H6_/6) × 100 [7]. I_AR_ and I_H2-H6_ indicated peak areas of aromatic protons in FITC and C2-C6 protons in the chitosan backbone, respectively.

### 2.4. Fluorescent Analyses of the CHI Oligomer and CHI Oligomer-FITC Complexes

To analyze the UV-Vis absorbance and photoluminescence (PL) properties of the CHI oligomer, aqueous solutions (0.1%–7% (*w*/*v*)) of the CHI oligomer in DW were prepared after stirring at room temperature for 1 h. The absorption spectra of the solutions were conducted on a UV-Vis spectrophotometer (UV-2450PC, Shimadzu Co., Kyoto, Japan) with a measurable range of 190–1100 nm and a resolution of 0.1 nm. The photoluminescence spectra of the solution were collected using a luminescence spectrophotometer (Varian Cary Eclipse, Varian, Palo Alto, CA, USA) equipped with a xenon flash lamp excitation source. The absorption spectra were obtained at 25 °C and the emission spectra of CHI oligomer and CHI oligomer-FITC were obtained at 475 nm and 520 nm, respectively, using an excitation wavelength at 395 nm with resolution of 1 nm and scan rate of 600 nm/min. Also, the changes in the fluorescent spectra of the CHI oligomer-FITC complexes with or without metal ions were measured using the same UV-Vis and luminescence spectrophotometers.

### 2.5. Adsorption Behaviors of Metal Ions onto the CHI Oligomer-FITC Complex

To examine applicability of the CHI oligomer-FITC complex as a colorimetric sensor, the reactions between the complex and the metal ions such as Na^+^, Cr^+^, Ni^+^, Sn^+^, Li^2+^, Mg^2+^, Al^2+^, Co^+^, Ni^2+^, Cu^2+^, Zn^2+^, Cd^2+^, Hg^2+^, Pb^2+^, and Fe^3+^ were monitored. The solutions containing metal ions (10^−3^ M, 0.1 mL) were added into the CHI oligomer-FITC complex solutions (1 mL). After that, the changes in color of the mixed solutions were checked after exposure to UV radiation (Ex. = 395 nm).

## 3. Results and Discussion

### 3.1. Structural and Fluorescent Analyses of the CHI Oligomer

**Figure 1:**
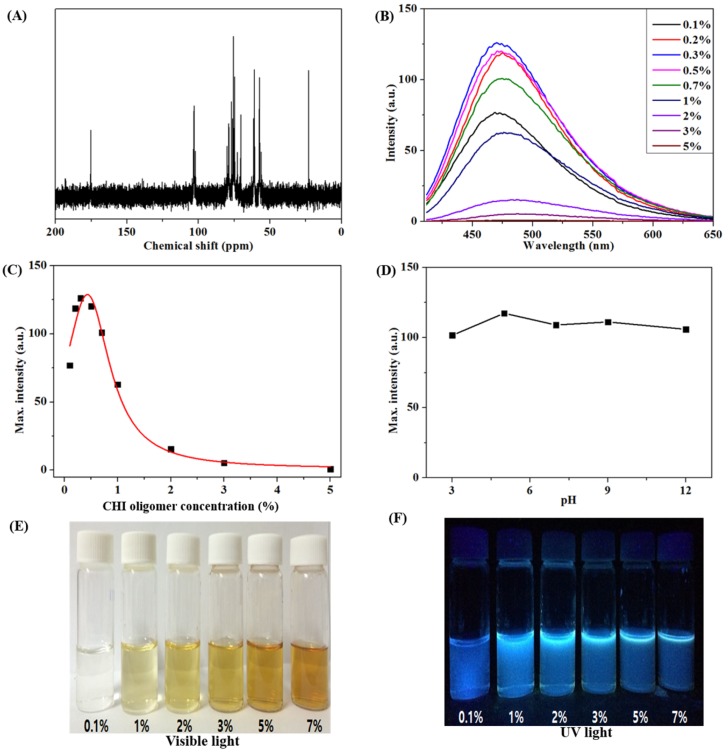
Analysis results of a CHI oligomer. ^13^C NMR spectra (**A**); PL spectra (**B**), change in fluorescent intensities at 470 nm by the concentration of the CHI oligomer (**C**); change in fluorescent intensities at 470 nm by pH conditions (**D**); images under visible (**E**) or ultraviolet light (Ex. = 395 nm) (**F**).

The low-molecular-weight CHI oligomer with a high water solubility showed unique structural and fluorescent characteristics. In general, fluorescence is caused by molecules with delocalized electrons at conjugated double bonds [14]. The CHI oligomer featured a molecular structure that had no delocalized electron, but the fluorescent property of the oligomer was observed by the fluorescence photometer and the naked eye. Recently, several polymers without conjugated double bonds were reported to exhibit fluorescence [15], and it turned out that the carbamato anion (NHCOO-) formed by the reaction between carbon dioxide and amine induced this phenomenon [16]. The ^13^C NMR spectrum in Figure 1A shows a typical carbamato anion (NHCOO-) peak in CHI oligomer at 174 ppm. It was induced by the reaction between the amino group in the CHI oligomer and carbon dioxide in air. The fluorescent intensity was monitored depending on the concentration of the CHI oligomer (Figure 1B,C). The excitation and emission wavelengths of the CHI oligomer were 400 and 470 nm, respectively.

The photoluminescence (PL) spectra revealed that the maximal CHI oligomer concentration showing the highest PL intensity was 0.3% (*w*/*v*). At 0.5% (*w*/*v*) and over, the PL intensities showed a tendency to decrease due to self-quenching among the CHI oligomer molecules [17]. The fluorescent pattern of the CHI oligomer was confirmed to be similar to that given in the results above under UV radiation (Ex. = 395 nm) (Figure 1E,F). In addition, observation at various pH values from 3 to 12 revealed that the PL intensities of the CHI oligomer were unaffected by the pH 3–12 conditions (Figure 1D).

### 3.2. Structural and Fluorescent Analyses of the CHI Oligomer-FITC Complexes

**Figure 2:**
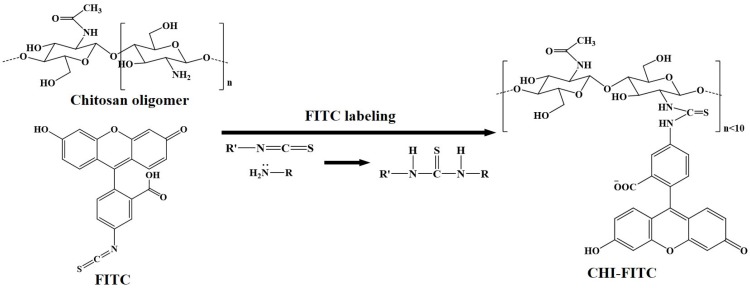
Schematic representation of a reaction mechanism between the CHI oligomer and FITC.

A reaction of a primary amine with an isothiocyanate (NCS), one of various amine modification methods, has been widely known to lead to a thiourea as its product [18]. Herein, to develop a sensitive and selective imaging probe that not only has high water solubility but is also immune to the pH conditions, the CHI oligomer–FITC complexes were prepared through a reaction of the CHI oligomer with FITC. The so-called “FITC labeling” reaction took place with the primary amine of the CHI oligomer and the NCS of FITC. The reaction mechanism is illustrated in Figure 2.

**Figure 3:**
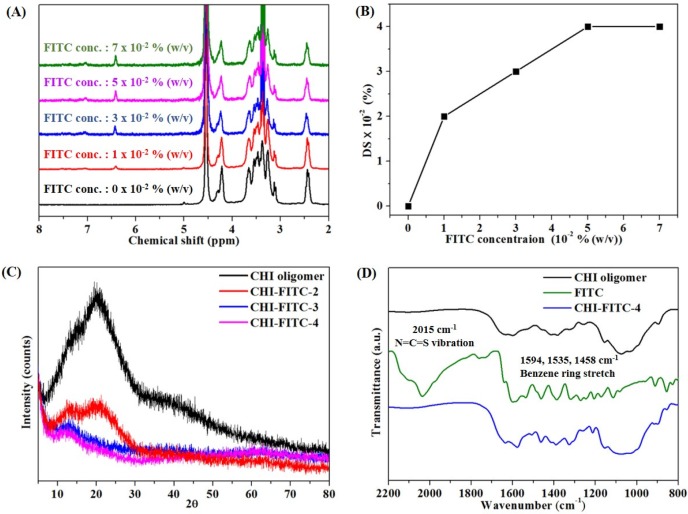
Results of the analysis of CHI-FITC complexes. ^1^H NMR spectra (**A**); DS (**B**); XRD spectra (**C**); FT-IR spectra (**D**).

**Figure 4:**
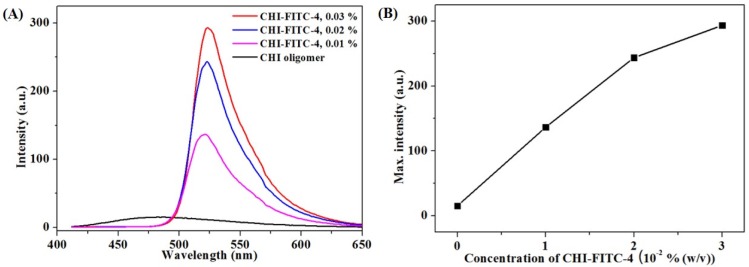
Results of the analysis of CHI-FITC-4 depending on the concentration. PL spectra (**A**); PL intensity at 520 nm (**B**).

The optimal condition of this reaction was confirmed by obtaining the ^1^H NMR, XRD, and FI-IR spectra [19,20,21]. The concentration of the CHI oligomer was fixed, and the concentration of FITC varied from 0 to 7 × 10^−2^% (*w*/*v*). The peaks at 6.5–8 ppm, indicating aromatic rings, were observed with an increase in the FITC concentration (Figure 3A). Also, the DS (%) was calculated by the proportion of areas of 6.5 to eight peaks corresponding to the nine protons of FITC to areas of three to four peaks corresponding to the six protons of the CHI oligomer (Figure 3B). The DS (%) values increased to four depending on the FITC concentration. Therefore, the sample with DS (%) = 4 was named as CHI-FITC-4. In the same way, the samples with DS (%) = 2 or DS (%) = 3 were named as CHI-FITC-2 or CHI-FITC-3, respectively. Figure 3C shows the XRD patterns for the CHI-FITC complexes. A spectrum of the CHI oligomer showed 2θ = 20° as a typical crystalline peak. However, according to the increase in the DS (%) value, the crystalline peak gradually decreased due to the introduction of a bulky FITC group. Due to the substitution of the amino group to thiourea by FITC, the steric hindrance between the CHI oligomers increased considerably, and hydrogen bonds were broken between the CHI oligomers. Also, through an FT-IR analysis, the peaks at 1458, 1535, and 1594 cm^−1^ related to the stretching vibration of the aromatic ring were clearly observed in the CHI-FITC-4 (Figure 3D). After the reaction of the CHI oligomer with FITC, a peak at 2015 cm^−1^ corresponding to the NCS vibration disappeared completely. Figure 3 verifies that the CHI oligomer–FITC complex was successfully synthesized. In addition, the fluorescent intensities of CHI-FITC-4 were evaluated from the PL spectra (Figure 4A,B), and the intensities were dependent on the concentration of CHI-FITC-4.

### 3.3. Colorimetric Sensing of CHI-FITC-4 against Metal Ions

**Figure 5:**
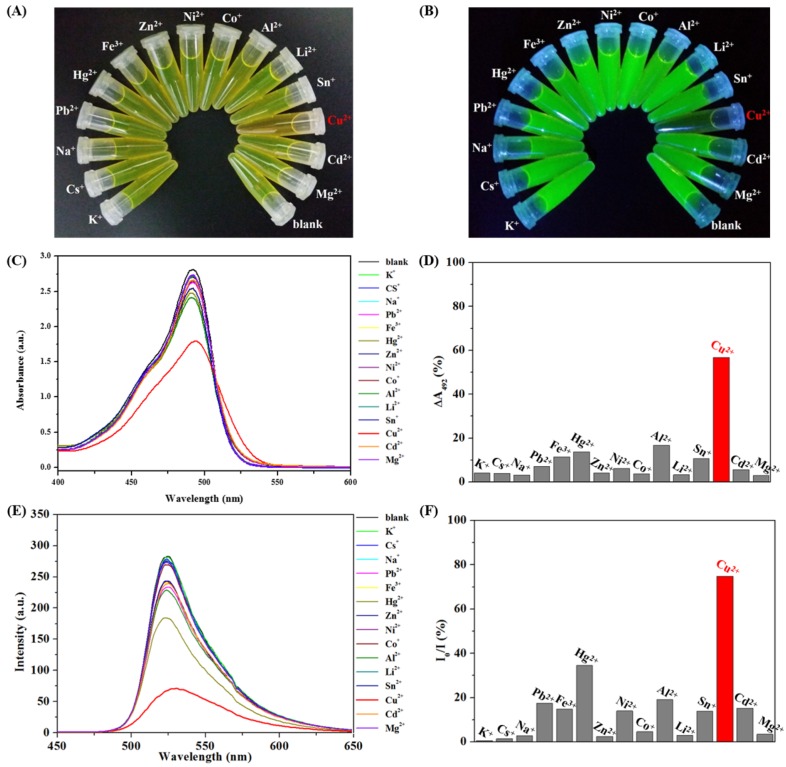
Colorimetric assay to sense metal ions using CHI-FITC-4. Images under visible (**A**) or ultraviolet radiation (**B**); UV-vis spectra (**C**); Change comparison of UV-vis intensity at 492 nm (**D**); PL spectra (**E**); Change comparison of PL intensity at 520 nm (**F**).

**Figure 6:**
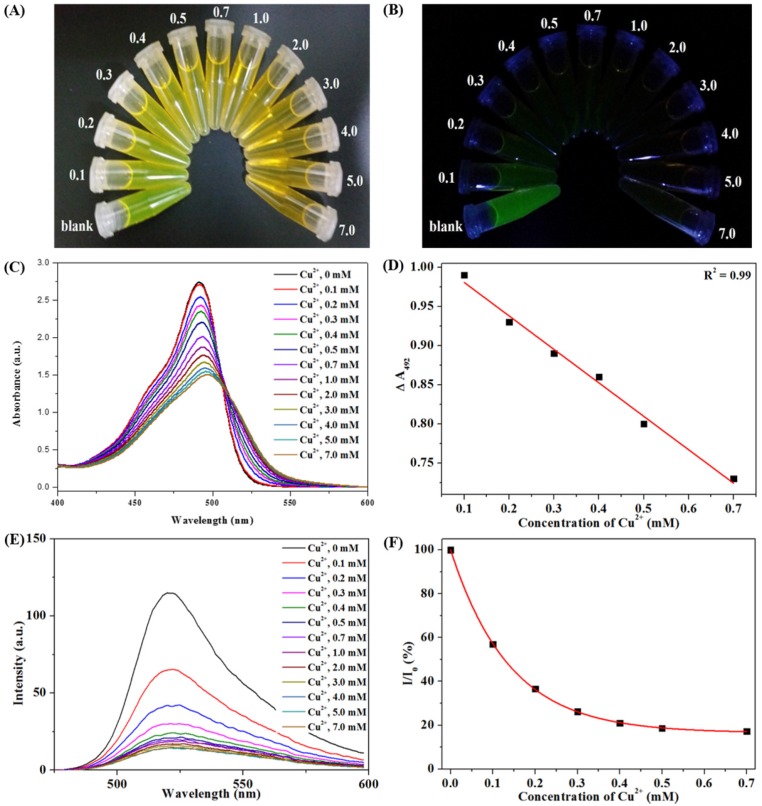
Colorimetric assay to sense Cu^2+^ using CHI-FITC-4. Images under visible (**A**) or ultraviolet radiation (**B**); UV-vis spectra (**C**); Change comparison of UV-vis intensity at 492 nm (**D**); PL spectra (**E**); Change comparison of PL intensity at 520 nm (**F**).

**Figure 7:**
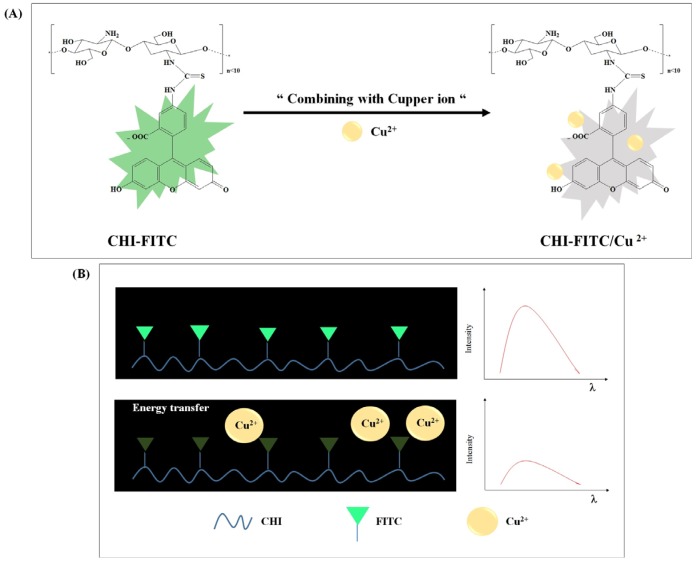
Schematic representation of the reaction mechanism between CHI-FITC and Cu^2+^ (**A**) and the energy transfer phenomenon (**B**).

Selectivity and sensitivity tests were carried out to assess the applicability of CHI-FITC-4 as a colorimetric sensor for metal ions. To begin, the selectivity of CHI-FITC-4 was investigated using metal ions such as Na^+^, Cr^+^, Ni^+^, Sn^+^, Li^2+^, Mg^2+^, Al^2+^, Co^+^, Ni^2+^, Cu^2+^, Zn^2+^, Cd^2+^, Hg^2+^, Pb^2+^, and Fe^3+^. Figure 5A,B show that the CHI-FITC-4 sample had selectivity for Cu^2+^. In particular, a decrease in the fluorescent intensity could be clearly distinguished from those of other metal ions under UV radiation (Ex. = 395 nm). As quantitative criteria, the UV-vis and PL spectra of CHI-FITC-4 with metal ions were monitored (Figure 5C–F). The absorbance (λ_max_ = 492 nm) and fluorescent intensity (λ_max_ = 520 nm) of CHI-FITC-4 with Cu^2+^ decreased to approximately 60% and 80%, respectively. According to the concentration range of 0.1–7.0 mM for Cu^2+^, a sensitive colorimetric assay of CHI-FITC-4 was implemented, and the color changes were observed under UV radiation (Ex = 395 nm) (Figure 6A,B). The UV-vis spectra indicated that the limit of detection (LOD) of CHI-FITC-4 for Cu^2+^ was close to 60 µM, and its correlation coefficient (*R*^2^) was 0.99 (Figure 6C,D). In addition, the fluorescent intensities at 520 nm of CHI-FITC-4 with Cu^2+^ displayed a tendency for decay according to the concentration (0.1–7.0 mM) of Cu^2+^ (Figure 6E,F). This phenomenon could be explained by the fluorescent quenching mechanism. The fluorescence intensity of CHI-FITC-4 decreased due to energy transfer resulting from the formation of a selective complex with Cu^2+^, as shown in Figure 7. The energy transfer occurred due to Cu^2+^ being located near the FITC group, resulting in a decrease in fluorescence (quenching) [22].

## 4. Conclusions

In this study, aqueous solutions of low-molecular-weight CHI oligomers were successfully prepared, and their structural and fluorescent properties were then meticulously observed using ^1^H NMR, ^13^C NMR, FT-IR, XRD, UV-Vis, and PL spectrometry. The presence of the carbamato anion (NHCOO-) as a fluorophore was verified as the origin of the fluorescent properties of the CHI oligomer. With respect to the concentration and pH of the aqueous solutions of the CHI oligomer, the optimal concentration of the oligomer was 0.3% (*w*/*v*), and the solutions were not affected in the range of pH of 3–12. Depending on the addition of the metal ions, the color changes of the synthesized CHI oligomer–FITC complex showed a remarkable difference. It had an excellent selectivity to detect copper ions (Cu^2+^), and its limit of detection (LOD) was 60 μM. When Cu^2+^ was combined with FITC, the energy transfer between FITC and Cu^2+^ led to fluorescent quenching of the CHI oligomer–FITC complex. In conclusion, the CHI oligomer–FITC complex has great potential as a promising colorimetric sensor to detect Cu^2+^.
